# Radiosensitization and Radioprotection by Curcumin in Glioblastoma and Other Cancers

**DOI:** 10.3390/biomedicines10020312

**Published:** 2022-01-28

**Authors:** Vasiliki Zoi, Vasiliki Galani, Pericles Tsekeris, Athanasios P. Kyritsis, George A. Alexiou

**Affiliations:** 1 Neurosurgical Institute, University of Ioannina, 45500 Ioannina, Greece; vasozoi95@gmail.com (V.Z.); thkyrits@uoi.gr (A.P.K.); 2 Department of Anatomy Histology-Embryology, School of Medicine, University of Ioannina, 45110 Ioannina, Greece; vgalani@uoi.gr; 3 Department of Radiation Oncology, University of Ioannina, 45110 Ioannina, Greece; ptsekeri@uoi.gr

**Keywords:** curcumin, cancer, radiation therapy, radiosensitizer

## Abstract

Radiation therapy plays an important role in almost every cancer treatment. However, radiation toxicity to normal tissues, mainly due to the generation of reactive free radicals, has limited the efficacy of radiotherapy in clinical practice. Curcumin has been reported to possess significant antitumor properties. Although curcumin can sensitize cancer cells to irradiation, healthy cells are much less sensitive to this effect, and thus, curcumin is thought to be a potent, yet safe anti-cancer agent. In this review, a summary of the role of curcumin as both a radiosensitizer and radioprotector has been presented, based on the most recent data from the experimental and clinical evaluation of curcumin in different cancer cell lines, animal models, and human patients.

## 1. Introduction

Radiation therapy (RT) plays a pivotal role in cancer treatment, along with surgery and chemotherapy. Radiotherapy induces DNA damage either directly by ionization or indirectly through the generation of reactive oxygen species (ROS) [1]. It has been used for over 100 years for the treatment of solid tumors, including cancers of the skin, breast, prostate, and brain, and has also been used to treat leukemia, gliomas, and lymphoma. Novel radiotherapy modalities, including proton and carbon ions, and stereotactic radiotherapy have also, gained the interest of scientists. Traditionally, RT has been delivered in a fractionated manner, and the total dose is broken up into daily doses five times a week. In some cases, however, accelerated RT has been found to increase the overall survival of patients and local control of tumors, particularly head and neck cancer [2,3].

Although RT remains the most effective non-surgical technique in the treatment of malignant tumors, radiation toxicity to normal tissues, different side effects affecting various organs such as skin, and the development of significant radioresistance in cancer cells are limiting its efficacy [4,5]. The development of radioresistance is associated with different mechanisms, including epigenetic alterations or the activation of survival signaling pathways [1].

Ionizing radiation causes damage to normal tissues primarily through the direct deposition of energy into vital macromolecules or the generation of reactive free radicals. [6] Those radicals react with biomolecules, like proteins and lipids, and cause oxidative damage in them [7]. Although fractionated irradiation in combination with improved treatment modalities has made radiotherapy safer than before, it is still important to broaden the therapeutic window between normal tissue damage and tumor suppression [8,9]. Two strategies are of clinical importance: radiosensitization of tumor cells without sensitizing normal cells and radioprotection of normal cells [10].

**Figure 1:**
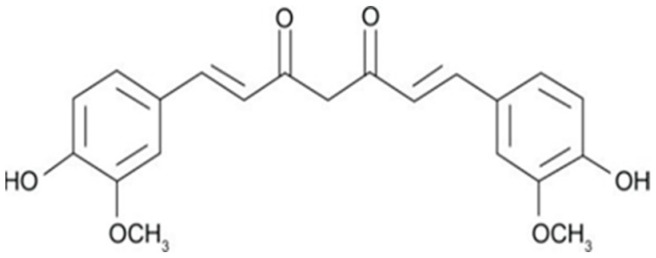
Chemical structure of curcumin.

Curcumin (Figure 1) (also known as diferuloylmethane) is extracted from the turmeric plant and possesses several health benefits, most of which have been attributed to its anti-inflammatory properties [11]. Curcumin has also been used in the management of various skin conditions, such as alopecia, skin infections, and atopic dermatitis [12,13]. In cancer treatment, curcumin has been studied alone and/or in combination with chemotherapeutic agents and radiation therapy [14,15]. Although curcumin can sensitize cancer cells to irradiation, healthy cells are much less sensitive to this effect and thus, curcumin is considered a potent, yet safe anti-cancer agent [16,17,18]. Moreover, curcumin has been found to possess radioprotective properties, since it can lessen inflammatory toxicities associated with radiotherapy, like dermatitis, mucositis, and myelosuppression [19,20]. In this review, we focus on this dual mode of action of curcumin and its analogs in relation to radiotherapy and have summarized the most recent evidence of its role as both a radiosensitizer and radioprotective agent.

## 2. Radiosensitization by Curcumin

Radiosensitizers are molecules that can amplify radiation-induced cellular damage [21]. Several radiosensitizers, including paclitaxel and cisplatin, have been studied with the aim to unravel their mechanism of action and the possible effect on normal cells surrounding the tumor. Most of those compounds, however, exhibit side effects that exclude them for use as a treatment option for all irradiated patients [22,23]. Moreover, resistance factors, such as the repair of radiation-induced DNA damage make the successful killing of all remaining cells capable of tumor regrowth almost inevitable [24].

**Figure 2:**
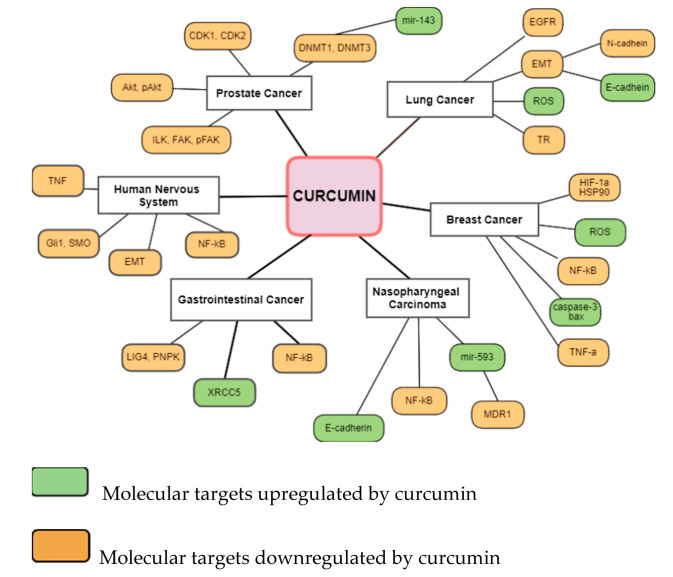
Molecular targets of curcumin contribute to radiosensitization in different cancers. Abbreviations: CDK: cyclin-dependent kinase; Akt: protein kinase B; ILK: integrin-linked kinase; FAK: focal adhesion kinase; DNMT: DNA methyltransferase; EGFR: endothelial growth factor receptor; EMT: epithelial–mesenchymal transition; ROS: reactive oxygen species; TR: trypanothione reductase; HIF-1a: hypoxia-inducible factor 1-alpha; HSP90: heat shock protein 90; NF-κB: nuclear factor-κB; bax: bcl-2-like protein 4, TNF-a: tumor necrosis factor-a; MDR1: multidrug resistance protein 1; XRCC5: X-ray repair cross-complementing 5; LIG4: ligase 4; PNPK: polynucleotide kinase/phosphatase; Gli1: glioma-associated oncogene homologue 1; SMO: smoothened.

Among the most thoroughly studied radiosensitizing agents is curcumin. Curcumin has been studied for its anti-cancer properties in different types of cancer and almost all evidence of radiosensitization come from laboratory data [11]. Curcumin can radiosensitize cells through different defined pathways. (Figure 2) Ionizing radiation has been reported to modify intracellular signaling mainly through modification of the activity of RAS-associated proteins, growth factors, and their receptors, like epidermal growth factor (EGFR), and transforming growth factor-β (TGF-β) [25]. Curcumin can suppress the gene expression of EGFR, and downregulate the TGF-β pathway, thus leading to inhibition of cancer-associated fibroblasts (CAF)-mediated cancer progression [26].

Ionizing radiation enhances the production of reactive oxygen species (ROS) that can cause DNA damage through the development of double-stranded breaks. After that, proteins related to DNA reparation, like DNA-dependent protein kinase are activated to act in favor of the DNA damage response pathway [27]. Curcumin can induce ROS generation and suppress DNA repair machinery, thus leading to increased radiation-induced cell death [28,29].

Apoptosis is the most common type of cell death induced by radiation in malignant tumors [30]. Although irradiation mostly induces the intrinsic apoptotic pathway, depending on both cell type and dose, the extrinsic apoptotic pathway (death receptor-mediated) can also, be involved [31]. Curcumin has been found to induce apoptosis through a series of actions on different signaling pathways, including upregulation of both the expression and activity of p53, regulation of the anti-apoptotic PI3K signaling, and suppression of the activity of NF-κB and COX-2 [32].

## 3. Curcumin as a Radiosensitizer in Various Malignant Tumors and Glioblastoma

In human non-small cell lung cancer A549 cells, curcumin (10 μΜ) has been shown to promote cell death following x-ray irradiation through inhibition of the epidermal growth factor receptor (EGFR)-associated pathway [33]. EGFR has been recently related to radioresistance in different tumors, however, the exact mechanism following this relationship requires further investigation [34]. In another study, A549 cells were exposed to irradiation to incite an epithelial-mesenchymal transition (EMT) model [35]. EMT plays a crucial role in metastasis which is characterized by the upregulation of mesenchymal molecular markers, like N-cadherin, and downregulation of epithelial molecular markers, including E-cadherin [36]. Curcumin was found to suppress radiation-induced EMT resulting in the inhibition of NSCLC migration and invasion [35].

When a metabolically stable analog of curcumin known as dimethoxycurcumin (DIMC), was used in combination with radiation, both an apoptotic and mitotic death in A549 cells was observed as a result of the inhibition of thioredoxin reductase activity. When thioredoxin reductase is inhibited, the oxidized form of thioredoxin accumulates in cells, resulting in increased oxidative stress. When combined with 4Gy radiation, DIMC (2.5 μΜ) significantly increased the levels of ROS, whereas a slowdown in DNA repair was also, observed [37]. In a xenograft model of A549 cells, curcumin-loaded nanoparticles enhanced the tumor growth inhibitory effect of radiation, mainly through induction of apoptosis [38].

Τhe radiosensitizing effect of curcumin on nasopharyngeal carcinoma (NPC) cells has been studied to unravel whether the exact mechanism is associated with multidrug resistance gene 1 (MDR1) and microRNA-593. At 10 µmol/L, curcumin upregulated the expression of miR-593, resulting in the depression of MDR1 expression, which may promote radiosensitivity of NPC cells. When curcumin (100 mg/kg) was given prior to 4 Gy irradiation in a transplanted tumor model in vivo, a significant tumor growth inhibition was observed in the irradiation plus curcumin group compared to the irradiation only group. [39] In another study, where human NPC cell line CNE-2 was used, curcumin enhanced radiosensitization through modulation of the circular RNAs (circRNAs) network. [40] CircRNAs are known to take part in the cellular irradiation response and some of them, including KIRKOS-71 and KIRKOS-73 have been studied as promising diagnostic radiotherapy biomarkers [41].

In three breast cancer cell lines (MCF10A, MCF7, and MDA-MB-231 BC) curcumin-loaded solid nanoparticles (2.5–10 μΜ) were used in combination with increasing doses of irradiation (2–9 Gy). Based on a transcriptomic and metabolomics study, the combination of curcumin and radiotherapy resulted in the deregulation of molecules involved in the induction of apoptosis, in the inflammatory process, in the cell cycle, and in tyrosine metabolism in both MCF7 and MDA-MB-231 BC cell lines. In the non-tumorigenic MCF10A cell line, the effect of the combination treatment was mostly related to lysine degradation and transcriptional misregulation [42]. In breast cancer stem cells, curcumin combined with glucose nanogold particles managed to significantly reduce radiotherapy resistance under hypoxic conditions. The molecular mechanism underlying this effect was found to be related to inhibition of the expression of both hypoxia-inducible factor 1-alpha (HIF-1a) and heat shock protein 90 (HSP90) proteins and increase in the levels of ROS [43].

In cervical cancer, curcumin has been studied as a potent mTOR inhibitor when given together with irradiation. When a human cervical cancer cell line (HeLa) was treated with increased curcumin concentrations followed by irradiation (2 Gy) using a linear accelerator, increased cell cytotoxicity was observed compared to treatment with irradiation or curcumin alone [44]. In a randomized control trial that included cervical carcinoma stage IIB–IIIB patients, the levels of survivin, which is an important anti-apoptotic protein, were assessed in patients receiving curcumin and radiation as opposed to those treated with irradiation alone. Decreased levels of this protein were observed in 75% of patients having received the combination, whereas, in the group treated with irradiation alone, 60% of patients showed increased levels of survivin [45].

In prostate cancer, the radiosensitizing effect of curcumin has been correlated to epigenetic activation of miR-143. Pretreatment of PC-3, DU145, and LNCaP cells with curcumin, prior to exposure to irradiation resulted in the reduction in the expression of prototypic DNA methyltransferase DNMT1 and DNMT3B, both of which contribute to the hypermethylation of the miR-143/miR-145 cluster. Furthermore, curcumin pretreatment increased radiation-induced apoptosis [46]. In another study, where DU145 and PC-3 cells were treated with low doses of curcumin (0.1–0.4 µg/mL) prior to irradiation with 1.65 J/cm^2^ visible light, it was observed that the combination resulted in significant tumor growth suppression, and both adhesion and migration were hindered [47].

Curcumin has shown significant radiosensitizing properties in tumors developed in the human nervous system. When LN229 and U251 glioma cells were treated with curcumin (20 μM) after exposure to γ-irradiation, the irradiation-stimulated epithelial-mesenchymal transition (EMT) process was suppressed through inhibition of the Hedgehog signaling pathway, which is characterized by an increase in E-cadherin levels. Moreover, combined treatment with curcumin and ionizing radiation reduced both the cell migration and invasion abilities of both cell lines. The same effect on the EMT process was observed when combination treatment was given in intracranial glioma models of nude mice [48]. In another study that included U87 and T98 human glioma cells, the combination treatment of curcumin and radiotherapy resulted in increased cytotoxicity and a more prominent G2/M arrest compared to individual treatment [49]. The synergistic effects of curcumin and radiotherapy were also validated in an orthotopic F98/FGT glioma-bearing rat model. Curcumin enhanced the effects of radiotherapy resulting in increased suppression of the growth of in situ brain tumors and transplanted glioma cells alike [50].

Several studies have shown the radiosensitizing effects of curcumin in gastrointestinal cancers. In five different human esophageal squamous cell lines ESCC-07, ESCC-12, ESCC-19, ESCC-27, and ESCC-31, curcumin increased radiation-induced apoptotic death primarily through inhibition of the NF-κB signaling pathway. Pretreatment with curcumin also suppressed tumor progression and decreased both the tumor volume and weight of ESCC-07 xenograft mice that were previously exposed to fractionated radiotherapy [51]. When low doses of curcumin (2.5 μΜ) were combined with radiation in human colon cancer HT-29 cells, a stronger inhibitory effect on cell proliferation was observed, due to modulation of expression of DNA repair-related genes, including Cyclin H (CCNH), DNA Ligase 4 (LIG4) and Polynucleotide Kinase 3′-Phosphatase (PNKP). When HT-29 bearing mice were treated with curcumin and irradiation, the combined treatment resulted in higher intratumoral apoptosis and suppression of neoplastic growth [52]. In human pancreatic cancer lines Panc-1 and MiaPaCa-2, curcumin enhanced radiation-induced apoptosis and increased the G2/M-fraction at the irradiation time point in both cell lines [53].

Curcumin has been reported to increase the radiosensitivity of renal ACHN cancer cells through suppression of the NF-κB signaling pathway and modulation of the associated protein levels, like COX-2 and Bcl-2. In ACHN tumor-bearing nude mice, curcumin and irradiation treatment resulted in decreased tumor volume and enhanced apoptotic death [54]. Inhibition of cell viability and clonogenic survival was observed in urinary bladder cancer T24 cells after treatment with curcumin (10 µM) in combination with radiation. This effect was attributed to the downregulation of miR-1246 expression, resulting in inhibition of the p53 nuclear transcription factor [55]. A summary of the most recent studies on the radiosensitizing effects of curcumin on different cancer models is depicted in Table 1.

## 4. Radioprotection by Curcumin

Radiotherapy plays an important role in cancer treatment. The fraction of all cancer survivors who received radiation increased from 24% in 2000 to 29% in 2020 [56]. Normal tissue toxicity is, however, a limiting factor for receiving sufficient doses of radiation to kill the tumor [57]. Common radiation toxicities include dermatitis, pneumonitis, myelosuppression, secondary tumors, mucositis, and skin irritation. Since most of these toxicities are inflammatory in nature, they are amenable to the anti-inflammatory properties of curcumin [58].

During radiotherapy, about 90% of patients experience acute skin reactions [59]. The mechanism of radiation-induced skin toxicities has been related to apoptosis and necrosis even weeks after irradiation. [60] In a recent study, where 40 rats were exposed to curcumin 1 day before irradiation to 3 consecutive days after irradiation, the levels of antioxidant enzymes, including catalase (CAT), superoxide dismutase (SOD), and malondialdehyde (MDA), were found to be considerably elevated after curcumin treatment, suggesting that curcumin reacted to the radiotherapy-induced oxidative damage [61]. The therapeutic effect of topical application of curcumin after gamma-radiation exposure was assessed in a mini-pig model. Curcumin was applied topically to the irradiated skin (200 mg/cm^2^) twice a day for 35 days. The curcumin-treated group showed inhibition of the irradiation-induced increases in NF-κB and COX-2 expression. Moreover, treatment with curcumin stimulated wound healing, thus improving the clinical appearance of the biopsy wound more quickly compared to the vehicle-treated group [62] (Table 2).

Radiation dermatitis is characterized by erythema, dry desquamation, ulceration and occurs in 90% of patients undergoing RT [63]. In a randomized, placebo-controlled, blinded study of 191 breast cancer patients, curcumin was applied topically on the irradiated skin daily for a week after irradiation completion. In patients with high breast separation (≥25 cm), who may have the worst skin reactions, curcumin was found to be effective in minimizing RT dermatitis and pain [64]. In another randomized, double-blinded, placebo-controlled trial of 686 breast cancer patients, however, in which patients received 500-mg capsules of curcumin three times daily for up to 1-week after radiation therapy, no significant reduction in RT dermatitis severity was observed compared to the placebo group [65] (Table 2).

Radiation-induced lung injury poses a great threat to patients exposed to total body irradiation. Pneumonitis usually appears some months after radiotherapy, whereas fibrosis may appear a few years later [66]. Curcumin has been found to mitigate both pneumonitis and fibrosis in rats through downregulation of the expression of IL-4, IL4Ra1, DUOX1, and DUOX2 [67]. Interleukin (IL)- 4 has been related to the development of radiation-induced inflammation, while dual oxidase 1 and 2 (DUOX1 and 2), are responsible for chronic H_2_O_2_ production [68,69]. The radio-protective effects of curcumin in this rat model were also attributed to its ability to attenuate macrophages as well as lymphocyte infiltration [67]. In another recent study, where curcumin was loaded in a mesoporous polydopamine nanoparticles (CMPN) system and administrated intratracheally to the lung of rats after exposure to 15 Gy 60Co γ-ray radiation on the chest area, a reduction in proinflammatory cytokines, and malondialdehyde (MDA), along with an increase in SOD was observed [70] (Table 2).

Oral mucositis (OM) is caused by injury to salivary glands and arterioles, followed by the release of ROS and inflammatory cytokines. Patients under head and neck radiotherapy are more susceptible to the development of OM, although the exact risk depends on multiple factors, including gender, age, tobacco use, and radiotherapy dosage [71]. In a recent clinical study, 50 patients under chemotherapy were divided into two groups according to their exposure or not to head and neck radiotherapy. All patients received 80 mg of curcumin nanomicelle capsules twice a day for 7 weeks. The results showed that in patients who were under chemotherapy and head and neck radiotherapy, curcumin administration managed to significantly decrease the OM severity [72]. Similar results were presented by Charantinath et al. who evaluated the efficacy and safety of a curcumin gel in the alleviation of OM in cancer patients undergoing radiochemotherapy [73]. The efficacy of curcumin in reducing OM in cancer patients has also been attested in a triple-blind, pilot randomized controlled trial, which included 74 head and neck cancer patients scheduled to receive RT. The use of 0.1% curcumin mouthwash managed to decrease the risk of getting the onset of OM by 50% [74] (Table 2).

Curcumin may also protect lymphocytes, the most RT-susceptible type of blood cells against genotoxicity. When 21 patients with differentiated thyroid carcinoma were treated with I-131 (activity 5.5 GBq), and then received curcumin at a dosage of 160 mg/day for 10 days, exhibited a significantly lower frequency of micronuclei in peripheral blood lymphocytes compared to the placebo group [75]. In another study, human peripheral blood lymphocytes (HPBLs) were treated with increased curcumin concentrations (0.125–50 µg/mL) prior to exposure to 3 Gy of γ-radiation. Curcumin pre-treatment inhibited the formation of different free radicals, including hydroxyl (OH), nitric oxide (NO), and 2,2′-diphenyl-1-picrylhydrazyl (DPPH) in a dose-dependent manner [76]. Similar results were observed in a study involving human blood cells obtained from healthy male donors when treated with curcumin-encapsulated liposomes at an optimal concentration of 30 μg/mL prior to exposure to gamma Cobalt-60 irradiation. A significant decrease in micronuclei formation was noted that was independent of the irradiation dose [77] (Table 2).

The radioprotective effect of curcumin on rat heart tissue has been investigated by Kolivand et al. Twenty rats were given 150 mg/kg curcumin for seven consecutive days after exposure to γ-rays (15 Gy). After 10 weeks, an increase in the infiltration of lymphocytes and macrophages was observed, as well as a decrease in the expression of Duox1 and Duox2. The levels of both IL-4 protein and its receptor were also, decreased in the curcumin-treated group. Those results signify a reduction in popular radiation-induced heart injury markers [78]. Oral administration of curcumin has also been related to decreased risk of developing irradiation-induced hepatic or liver damages. Essawy et al. found that curcumin treatment shows hepatoprotective effects against radiation-induced hepatotoxicity in rats, through regulation of the therapeutic targets CYP2E1, Nrf2, and NF-κB, whereas Li et al. found that curcumin treatment prior to radiation can prevent liver damages, mainly through the modulation of the NF-κB pathway and reduction of oxidative stress (upregulation of SOD, CAD and GSH levels in the curcumin-treated group) [79,80] (Table 2).

## 5. Conclusions and Future Perspectives

A substantial volume of scientific evidence exists that curcumin acts as both a radiosensitizer to tumors and radioprotector to normal cells. Moreover, several beneficial properties of curcumin, including ease of oral administration, its lack of systemic toxicity, and low cost, make this natural polyphenol a promising adjuvant agent for the treatment of various human cancers, in combination with standard radiation therapy. Exploitation of the radiosensitizing potential of curcumin could not only result in a better clinical outcome, particularly in cases where significant radioresistance to standard therapy has been observed, but also, in the reduction of the radiation doses that are required for the achievement of therapeutic effects. That can, in turn, lower both the extent and severity of adverse effects related to radiotherapy.

Mechanistically, the radiosensitizing effects of curcumin are achieved via modulation of different molecular targets which are involved in the proliferation, angiogenesis, apoptosis, and metastasis of cancer cells. The exact mechanism of action highly depends on the malignant cell type, and the timing of curcumin treatment. In most cases, pretreatment with curcumin before exposure to irradiation is required in order to achieve the maximum antitumor activity. In terms of radioprotection, the anti-inflammatory nature of curcumin has been the key to understanding this module of action, since most adverse effects related to RT, including dermatitis, oral mucositis, and pneumonitis are inflammatory in nature. However, it is evident that the molecular mechanisms underlying the radioprotective and radiosensitizing effects of curcumin should be fully understood in order to develop a novel combined radiotherapeutic strategy for cancer patients.

A factor that has still limited the clinical value of curcumin is its low bioavailability. Curcumin exhibits poor absorption, extensive metabolism, and rapid elimination from the body. Even when administrated at doses of 12 g/day, the bioavailability of curcumin remains distinctly poor [81]. However, modern strategies have been developed that aim to increase the limited bioavailability of curcumin (e.g., liposomal, molecular analogs, and conjugated forms). In that turn, the encapsulation of this compound into nanosized carriers has been shown to improve the direct delivery of curcumin to tumor sites considerably [82]. When a water-soluble curcumin formulation that consisted of curcumin, cellulosic derivatives, and a widely used hydrophilic carrier was made, a 46-fold increase in its oral absorption was observed [83]. Other strategies to help overcome the issue of low bioavailability have been reported by several scientific groups. Singh et al. developed a silica nanoparticle–CUR complex conjugated with hyaluronic acid that enhanced curcumin cytotoxicity on COLO-205 cancer cells, and increased its stability and uptake, as well [84]. Jyoti et al. showed that chitosan microspheres of CUR improved solubility and increased its cytotoxicity in HT-29 cells, [85] whereas Shinde and Devarajan developed a docosahexaenoic acid-mediated microemulsion of curcumin and showed that it can effectively release curcumin to the brain and inhibit the proliferation of human glioma U-87MG cells [86].

Although the radiosensitizing effect of curcumin has been widely explored by different scientific groups in several cancer models, curcumin-related oncological clinical studies listed in clinicaltrails.gov are also worth mentioning. For example, in a randomized, double-blind, placebo-controlled trial curcumin was given to prostate cancer patients with intermittent androgen deprivation resulting in a decrease in the levels of prostate-specific antigen (PSA) (NCT03211104) [87]. Topical application of curcumin showed a significant therapeutic effect in patients with oral submucous fibrosis particularly in combination with triamcinolone and hyaluronidase [88]. In a multi-site, randomized, placebo-controlled, blinded study of 191 breast cancer patients, topical application of curcumin was found to help reduce radiotherapy-associated dermatitis and pain [64]. There is also, accumulating evidence to suggest the potential anti-neoplastic activity of curcumin against HNCs [89,90].

Furthermore, the protective action of curcumin against radiation-induced damages on normal tissues is of significant clinical value. In that respect, the use of modern strategies to increase bioavailability is extremely important. When curcumin-encapsulated liposomes were prepared using commercial phosphatidylcholine, an enhanced radioprotective effect against genotoxicity caused by Gamma Cobalt-60 irradiation in human blood cells was observed [77]. Since curcumin is a safe and highly tolerable natural compound, it could be administrated in combination with traditional radiotherapy to improve the clinical outcome of cancer patients in the future. However, clinical trials in humans are needed to fully assess the radiosensitizing and radioprotective properties of this compound.

## Figures and Tables

**Table 1:** Radiosensitizing effects of curcumin on different cancer models.

Cancer Site	Subject	Curcumin Dosage	Effect	Ref.
Lung	Human A549 cells	10 μΜ	Ιncrease in IR-induced reduction of cell viability via inhibition of EGFR protein	[33]
	Human A549 cells	5–20 μΜ	Inhibition of migration, invasion through suppression of radiation-induced EMT	[35]
	Xenograft model of A549 cells	20 uM	Induction of apoptosis	[38]
Nasopharynx	Human nasopharyngeal carcinoma (NPC) cells	10 μΜ	Increase in radiosensitivity through depression of MDR1 expression	[39]
	Human NPC cell line CNE-2	20 μΜ	Increase in IR-induced cell death through modulation of circRNAs	[40]
Breast	Human MCF10A, MCF7 and MDA-MB-231 BC cells	2.5–10 μΜ	Deregulation of molecules involved in the induction of apoptosis, in the inflammatory process, in the cell cycle, and tyrosine metabolism	[42]
	Breast cancer stem cells	30 μΜ	Reduction of RT resistance through inhibition of HIF-1a, HSP90	[43]
Prostate	Human PC-3, DU145, and LNCaP cells	30 μΜ	Increase in IR-induced apoptosis/reduction in the expression of DNMT1 and DNMT3B	[46]
	DU145 and PC-3 cells	0.1–0.4 µg/mL	Tumor growth suppression, decreased invasion, and migration	[47]
Cervix	Human HeLa cells	40 μΜ	Increased cell cytotoxicity of the combination	[44]
	Cervical carcinoma stage IIB–IIIB patients	4 g/day	Decreased levels of surviving	[45]
CNS	LN229 and U251 glioma cells	20 μΜ	Reduction in cell migration and invasion/inhibition of the Hedgehog signaling pathway	[48]
	U87 and T98 human glioma cells	10–20 μΜ	Increased cytotoxicity and G2/M arrest	[49]
	Orthotopic F98/FGT glioma-bearing rat model.	5-20 μΜ	Suppression of the growth of in situ brain tumors	[50]
Esophagus	Human ESCC-07, ESCC-12, ESCC-19, ESCC-27 and ESCC-31 cell lines	10 μΜ	Increase in IR-induced apoptosis/Inhibition of Nf-Kb signaling	[51]
	ESCC-07 xenograft mice	10 μΜ	Decrease in tumor volume and weight	[51]
Colon	Human colon cancer HT-29 cells	2.5 μΜ	Inhibition of cell proliferation/modulation of expression of DNA repair-related genes	[52]
	HT-29 bearing mice	2.5 μΜ	Intratumoral apoptosis and suppression of neoplastic growth	[52]
Pancreas	Human Panc-1 and MiaPaCa-2 cells	6 or 12 μΜ	Increased cytotoxicity and G2/M arrest	[53]
Kidney	Renal ACHN cancer cells	5–80 μΜ	Increased cell death, suppression of the NF-κB signaling pathway	[54]
	ACHN tumor-bearing nude mice	5–80 μΜ	Decrease in tumor volume increased apoptosis	[54]
Urinary Bladder	Urinary bladder cancer T24 cells	10 μΜ	Inhibition of p53 nuclear transcription factor	[55]

**Table 2:** Recent studies on radiotherapy adverse reactions prevention/management with curcumin. ↑, upregulation; ↓, downregulation.

Adverse Reaction	Subject	Curcumin Dosage	Mechanism/Conclusion	Ref
Acute skin reactions (prevention)	40 rats	150 mg/kg 1 day before to 3 days post-radiation	↑ antioxidant enzymes (CAT, SOD, MDA)	[61]
Acute skin reactions (management)	mini-pig model	200 mg/cm² twice a day for 35 days after RT	↓ NF-κB and COX-2 expression, ↑ wound healing	[62]
Radiation dermatitis (prevention)	191 breast cancer patients	Curcumin gel 3 times daily for 1 week after RT	↓ RDS and Pain scores in patients with high breast separation (≥25 cm)	[64]
Radiation dermatitis (prevention)	686 breast cancer patients	500 mg three times daily for 1 week after RT	No sig. difference between curcumin and placebo in RDS	[65]
Radiation pneumonitis/fibrosis	20 rats	150 mg/kg for 4 days before and 6 consecutive days after RT	↓ IL-4, IL4Ra1, DUOX1 and 2 expression	[67]
Radiation pneumonitis	20 rats	2 mg i.t. 5 h pre-irradiation	↓ proinflammatory cytokines, MDA, and ↑ SOD expression	[69]
Oral mucositis (treatment)	50 head and neck cancer patients	80 mg of curcumin nanomicelle capsules twice a day for 7 weeks	↓ OM severity and pain	[72]
Oral mucositis (treatment)	40 patients with OM	Gel containing 10 mg of curcumin, 3 times a day for 2 weeks	↓ OM severity	[73]
Lymphocytes genotoxicity (treatment)	21 patients with differentiated thyroid carcinoma (DTC)	160 mg/day for 10 days post-RT	↓ frequency of micronuclei in peripheral blood lymphocytes	[75]
Lymphocytes genotoxicity (prevention)	Human peripheral blood lymphocytes (HPBLs)	0.125–50 µg/mL prior to RT	↓ formation of OH, NO, DPPH, micronuclei	[76]
Heart tissue toxicity (prevention)	20 rats	150 mg/kg curcumin for 7days after RT	↓ Duox1 and Duox2, IL-4 protein and its receptor ↑ infiltration lymphocytes, macrophages	[78]
Hepatic toxicity (prevention)	20 rats	100 mg/kg orally for 21 days before RT	Regulation of Nrf2, mir-122, Ca^2+^ level, NF-κB	[79]
Liver toxicity (treatment)	30 rats	30 mg/kg for 2 weeks once a day post-RT	↑ SOD, CAD, GSH, Bcl-2 ↓ TNF-α, IL-1β, IL-6, ↓ p-NF-κB/NF-κB	[80]
